# Dietary menhaden fish oil supplementation suppresses lipopolysaccharide-induced neuroinflammation and cognitive impairment in diabetic rats

**DOI:** 10.1080/13880209.2024.2351933

**Published:** 2024-05-16

**Authors:** Nurina Titisari, Ahmad Fauzi, Intan Shameha Abdul Razak, Mohd Hezmee Mohd Noor, Nurdiana Samsulrizal, Hafandi Ahmad

**Affiliations:** a Department of Veterinary Preclinical Sciences, Faculty of Veterinary Medicine, Universiti Putra Malaysia, Selangor, Malaysia; b Department of Veterinary Physiology, Faculty of Veterinary Medicine, Universitas Brawijaya, East Java, Indonesia; c Department of Clinical Pathology, Faculty of Veterinary Medicine, Universitas Brawijaya, East Java, Indonesia; d Faculty of Applied Sciences, Universiti Teknologi MARA, Shah Alam, Malaysia; e Institute of Tropical Agriculture and Food Security, Universiti Putra Malaysia, Selangor, Malaysia

**Keywords:** Brain, hyperglycaemia, interleukin, neurodegenerative, streptozotocin, tumour necrosis factor

## Abstract

**Context:**

Menhaden fish oil (FO) is widely recognized for inhibiting neuroinflammatory responses and preserving brain function. Nevertheless, the mechanisms of FO influencing brain cognitive function in diabetic states remain unclear.

**Objective:**

This study examines the potential role of FO in suppressing LPS-induced neuroinflammation and cognitive impairment in diabetic animals (DA).

**Materials and methods:**

Thirty male Wistar rats were divided into 5 groups: i) DA received LPS induction (DA-LPS); ii) DA received LPS induction and 1 g/kg FO (DA-LPS-1FO); iii) DA received LPS induction and 3 g/kg FO (DA-LPS-3FO); iv) animals received normal saline and 3 g/kg FO (NS-3FO) and v) control animals received normal saline (CTRL). Y-maze test was used to measure cognitive performance, while brain samples were collected for inflammatory markers and morphological analysis.

**Results:**

DA received LPS induction, and 1 or 3 g/kg FO significantly inhibited hyperglycaemia and brain inflammation, as evidenced by lowered levels of pro-inflammatory mediators. Additionally, both DA-LPS-1FO and DA-LPS-3FO groups exhibited a notable reduction in neuronal damage and glial cell migration compared to the other groups. These results were correlated with the increasing number of entries and time spent in the novel arm of the Y-maze test.

**Discussion and conclusion:**

This study indicates that supplementation of menhaden FO inhibits the LPS signaling pathway and protects against neuroinflammation, consequently maintaining cognitive performance in diabetic animals. Thus, the current study suggested that fish oil may be effective as a supporting therapy option for diabetes to avoid diabetes-cognitive impairment.

## Introduction

Long-term high glucose concentrations in the blood, referred to as hyperglycaemia, have been linked to a variety of organ and tissue damage, including the brain tissue (Berbudi et al. 2020). The necessity of glucose as the principal energy source for the normal functioning of the mammalian brain is indisputable (Takada et al. 2021). Glucose regulation is extremely strict, and if blood sugar levels are not treated or maintained, it can cause various physiological and pathophysiological changes leading to neurological disorders (Luna et al. 2021). In fact, a study claimed that neurodegenerative diseases and neuroinflammation could be controlled by managing blood glucose levels (Hung et al. 2022). As several theories have pointed out, there is a strong link between diabetes, brain inflammation, and the risk of developing neurodegenerative disorders (De Felice and Ferreira 2014; Pugazhenthi et al. 2017; Rom et al. 2019; De Sousa et al. 2020).

It is widely recognized that diabetic encephalopathy, a condition characterized by brain injury caused by diabetes mellitus, has the potential to impact the central nervous system, leading to cognitive impairment and motor dysfunctions (McCrimmon et al. 2012; Muramatsu 2020). The exact mechanism underlying this condition is complex and still not completely comprehended. However, a previous study reported that diabetic encephalopathy is associated with the presence of glucolipotoxicity, chronic inflammation, and oxidative stress burden (Díaz-Gerevini et al. 2019). Meanwhile, brain cells are vulnerable to oxidative stress, and the reactive oxygen species (ROS) generated in the brain may contribute to various neurodegenerative disorders (Anwar 2022). Therefore, it is essential to maintain blood sugar levels to avoid brain inflammation to prevent or slow down diabetic encephalopathy progression.

Generally speaking, insulin has served as the primary treatment for regulating glucose levels in the bloodstream (Mesa 2015). Regrettably, insulin has unavoidable side effects, including the destruction of pancreatic cells (Harrison 2021), the occurrence of severe hypoglycemia (Karges et al. 2017), and the disruption of brain insulin signaling (Crabtree et al. 2016). Furthermore, it has been recognized that consuming an adequate amount of fish oil, which is high in omega-3 fatty acids, helps to preserve the brain’s insulin signaling pathways (Bhatia et al. 2011), prevent insulin resistance (Agrawal and Gomez-Pinilla 2012), inhibit neuroinflammation and cognitive dysfunction (Lu et al. 2010; Devassy et al. 2016). While numerous studies have demonstrated the evidence of omega-3 fatty acid’s ability to successfully decrease brain inflammation and disturbance, its effectiveness in restoring blood glucose levels in individuals with diabetes is still uncertain and inconsistent. Several studies have suggested that omega-3 fatty acids have antidiabetic properties (Iwase et al. 2015; Laubertová et al. 2017). However, other studies have reported either less or no impact (Brown et al. 2019; Gao et al. 2020), or even an elevated risk of developing diabetes (Hu et al. 2022). Additionally, prolonged hyperglycaemia will promote abnormal expression of apoptosis-associated genes and inflammatory cytokines expression, leading to the initiation of neuronal death (Wang et al. 2021). This implies that keeping normal blood sugar levels is beneficial for maintaining good brain function.

To the best of our knowledge, the anti-inflammatory and neuroprotective potential of dietary menhaden fish oil supplementation associated with diabetes has not yet been explored. Thus, our objective was to examine the impact of fish oil supplementation on cellular and molecular changes in the brain, as well as cognitive performance in diabetic rats with LPS-induced brain inflammation. It has been established that the LPS induces systemic neuroinflammation, resulting in cognitive impairment (Batista et al. 2019). As a result, the systemic inflammatory response increases the synthesis and release of cytokines into the circulatory system, which may impact brain function (Perry 2004).

## Materials and methods

### Animals

Wistar rats (*n* = 30, male, 8 weeks) were purchased from Prima Nexus company, Malaysia and Anilab company (Indonesia). Rats were acclimatized for seven days and were given feed and water *ad libitum*. Rats were maintained in group cages (*n* = 3/cage) with ambient temperatures of around 20-26 °C. All animal procedures and experiments handling were performed according to the protocol of the Institutional for Animal Care and Use Committee (IACUC), Universiti Putra Malaysia, Selangor, Malaysia (UPM/IACUC/AUP-R017/2022) and also approved by the Research Ethics Committee of Universitas Brawijaya, Malang, Indonesia (070 KEP-UB-2022).

### Experimental induction of diabetes

Streptozotocin (STZ) (Cat no: SC-200719) was purchased from Santa Cruz Biotechnology (Aspire Biosains PLT, Malaysia). In the fasting condition, the animals were injected with STZ at 45 mg/kg/day (i.p) in 0.5 mL of 10 mM citrate buffer (pH 5.5) for three days continuously. The blood glucose was measured using a blood glucometer (Gluco Dr, South Korea) from the withdrawn tail blood vein. Animals were considered as hyperglycaemia or diabetic state if the blood glucose level at or more than 250 mg/dL (Gholamhosseinian et al. 2020).

### Experimental induction of neuroinflammation

Lipopolysaccharide (LPS) (Cat no: L2630) was purchased from Sigma-Aldrich Company (Aspire Biosains PLT, Malaysia). The animals were injected with a dilution of LPS (250 µg/kg) and normal saline by intraperitoneal (i.p) injection for seven days (Mahdi et al. 2019).

### Fish oil treatment

Menhaden fish oil (Cat no: F8020) was purchased from Sigma-Aldrich Company (Aspire Biosains PLT, Malaysia). The fatty acid composition of menhaden fish oil is shown in Table 1.

**Table 1.** Fatty acid composition of menhaden fish oil (sigma aldrich, USA).

Fatty acids compositions	Percentage (%)
Myristic acid (C14:0)	6% − 9%
Palmitic acid (C16:0)	15% − 20%
Palmitoleic acid (C16:1)	9% − 14%
Stearic acid (C18:0)	3% − 4 %
Oleic acid (C18:1)	5% −12%
Linoleic acid (C18:2)	< 3%
Linolenic acid (C18:3)	< 3%
Octadecatetraenoic acid (C18:4)	2% − 4%
Arachidonic acid (C20:4)	< 3%
Eicosapentaenoic acid (C20:5)	10% − 15%
Docosahexaenoic acid (C22:6)	8% − 15%
Unidentified fatty acids	20%

### Experimental design

A total of eighteen (*n* = 18) adult Wistar rats were injected with STZ to develop hyperglycaemia for three days. Next, these animals were induced with LPS for brain inflammation development for seven days. The animals were then randomly divided into three groups; i) diabetic animals with LPS induction (*n* = 6; DA-LPS); ii) diabetic animals with LPS induction and treated with 1 g/kg fish oil (*n* = 6; DA-LPS-1FO); iii) diabetic animals with LPS induction and treated with 3 g/kg fish oil (*n* = 6; DA-LPS-3FO). Another two groups of animals were selected as control; iv) animals received normal saline and treated with 3 g/kg FO (*n* = 6; NS-3FO) and v) control animals received normal saline (*n* = 6; CTRL). After 30 days of fish oil supplementation by oral gavage, all animals were assessed for cognitive performance using a Y-maze test. At the end of the test, all animals were euthanized by injection of sodium pentobarbital at 100 mg/kg and brain organs were collected for histological examination and analysis of inflammation markers.

### Cognitive performance by Y-maze test

The Y-maze apparatus was constructed from acrylic plastic and consisted of three arms: start arm, familiar arm, and novel arm, with an angle of 120° between each two arms. Each arm has a similar measurement: 60 cm in length, 15 cm in width, and 23 cm in heigh. The Y-maze test had two trials separated by an interval of 60 min. In the first trial, the animal was placed individually in the start arm for a duration of 10 min, with the novel arm being blocked. Subsequently, the animal was extracted from the Y-maze equipment and allowed to rest for a duration of 60 min. In the second trial, the animal had unrestricted access to all three arms and spent 5 min exploring the novel arm, which was not obstructed. Trials were documented through the utilization of a camera mounted on the ceiling, and subsequently, the recordings were reviewed to determine the quantity of entries and the duration spent in the novel arm. The higher cognitive performance of animals is demonstrated by the greater number of entries and increased time spent in the novel arm (Hafandi et al. 2014; Sopian et al. 2015).

### Relative cerebrum weight

The rat brain organ was isolated from the skull and washed with saline solution to remove excess blood. Later on, the weight of the cerebrum part was recorded using digital weight scale. The relative organ weights of the animals were calculated as:

$$\text{Relative cerebrum weight }\left(g/100\text{ g}\right):\frac{\text{Total cerebrum weight}}{\text{Final body weight}}\times 100$$

### Brain histology analysis

The left hemisphere of the cerebrum was thereafter preserved in a solution of neutral buffered formalin (NBF) at a concentration of 10% for a minimum duration of 24 h. Following fixation, the tissue samples were adjusted to the appropriate size and orientation and then inserted into the organ cassette. The samples were further dehydrated by subjecting it to a sequence of alcohol concentrations (70%, 80%, 85%, 90%, 95%, 100% ethanol). Samples were then treated with xylol to remove any remaining impurities, followed by paraffinization with paraffin. Finally, the samples were embedded and sectioned. The paraffin sections, which had a thickness of 4 μm, were subsequently treated to remove the paraffin and then stained using the hematoxylin-eosin (HE) method. The Optilab Camera Microscope (Software Image Raster) was used to capture photomicrograph images at magnifications of 10Ó¿ and 40Ó¿ objective lens magnification.

### Brain inflammation markers analysis

The flow cytometry approach was used to evaluate the expression of tumour necrosis factor-α (TNF-α) and interleukin-6 (IL-6) cytokines in the cerebrum homogenates. The right hemisphere of the brain was crushed in 1 mL phosphate-buffered saline (PBS) solution. The specimen mixture was inserted into the microtube and underwent centrifugation at a speed of 2500 revolutions per min (rpm) for 5 min at a temperature of 10 °C. Following the removal of the supernatant, 50 µL of fixation buffer (Cat no: 42001, Biolegend) was introduced and left to incubate at a temperature of 4 °C in a dark environment for a duration of 20 min. Subsequently, 500 µL of intracellular staining permeabilization wash solution (Catalogue number: 421002, Biolegend) was introduced, followed by centrifugation at 2500 rpm for 5 min at 10 °C. The supernatant was discarded, and the pellet was treated with 50 μL of antibodies TNF-α (Cat no: TN3-19.12, BioLegend) or IL-6 (Cat no: bs-0379R, Bioss antibodies). The mixture was then kept at a temperature of 4 °C in a dark environment for 20 min. Following the incubation period, 400 μL of PBS was added to each sample, which was subsequently placed into a flow cytometry cuvette for analysis.

### Statistical analysis

The collected data were analyzed using SPPS 20.0 software. The results were provided as mean ± SEM, except for the study of brain histology. A one-way ANOVA was used to compare all groups, followed by the Tukey *post hoc* test. The P value at *p* < 0.05 was considered to be a significant difference between groups.

## Results

### Effects of fish oil on blood glucose level of diabetic animals with LPS induction

Table 2 displays the blood glucose levels of different groups of animals. All diabetic rats that were induced with LPS (DA-LPS; DA-LPS-1FO and DA-LPS-3FO) expressed high blood glucose levels (>250 mg/dL) on day 3. Further, on the 30 days following the injection of STZ, all diabetic animals showed a reduction in blood glucose levels. Still, the blood glucose levels of the DA-LPS group remained above 250 mg/dL (259 ± 54.40), showing that the animals were still in a diabetic state. While the blood glucose levels of CTRL and NS-3FO (88.83 ± 8.73 and 68.67 ± 4.29, respectively) are not relatively similar to those of DA-LPS-1FO (135.17 ± 30.91). Nevertheless, both DA-LPS-1FO and DA-LPS-3FO groups showed a significant decrease in comparison to the DA-LPS group.

**Table 2.** The blood glucose levels (mg/dL) of different groups of animals after 3 and 30 days.

Groups	Blood glucose levels (mg/dL)	
	After 3 days	After 30 days
DA-LPS	413.57 ± 42.97***	259 ± 54.40**
DA-LPS-1FO	331.33 ± 14.7***	135.17 ± 30.91
DA-LPS-3FO	359.44 ± 14.87***	87.5 ± 15.23
NS-3FO	89.44 ± 21.59	68.67 ± 4.29
CTRL	98.89 ± 18.90	88.83 ± 8.73

Values are the mean ± SEM (*n* = 6) for each group. ** Significant difference (*p* < 0.01), *** significant difference (*p* < 0.001) compared to the CTRL group, using one-way ANOVA followed by the Tukey test.

### Effects of fish oil on cognitive performance of diabetic animals with LPS induction

**Figure 1.**
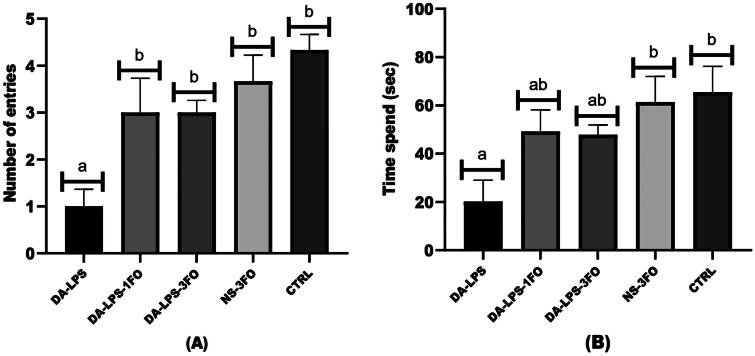
The Y-maze performance of diabetic animals with LPS and fish oil supplementation; (A) the number of entries and (B) The time spent in the novel arm. The result is expressed as means ± SEM. *significant difference (*p* < 0.001) compared to the CTRL group, using one-way ANOVA followed by the tukey test.

Figure 1 displays the outcome of cognitive performance as assessed by the Y-maze test. Diabetic animals with LPS induction (DA-LPS group) had the lowest number of novel arm entries and time spent in the novel arm compared to the NS-3FO and CTRL groups. On the other hand, the groups DA-LPS-1FO and DA-LPS-3FO exhibited a significant rise in the number of entries into the novel arm and the duration of time spent in the novel arm after three weeks of administering fish oil (at doses of 1 and 3 g/kg) in comparison to the DA-LPS group (as seen in Figure 1-A and 1-B).

### Effects of fish oil on brain relative weight and histopathology of diabetic animals with LPS induction

The cerebrum weight did not differ among the treatments, while the relative cerebrum weight of the DA-LPS group was significantly higher (*p* < 0.05) compared with the CTRL and NS-3FO groups (Table 3). Likewise, the DA-LPS-1FO showed significant differences in relative cerebrum weight when compared to both the normal and fish oil control rats (*p* < 0.05). Meanwhile, the DA-LPS-3FO group did not exhibit any statistically significant differences (*p* > 0.05) in terms of the relative weight of the cerebrum compared to the NS-3FO and CTRL groups.

**Table 3.** The weight and relative weight of the cerebrum between different groups.

Groups	Cerebrum weight (g)	Relative weight (g/100g)
DA-LPS	1.24 ± 0.054	0.60 ± 0.02*
DA-LPS-1FO	1.31 ± 0.04	0.59 ± 0.05*
DA-LPS-3FO	1.30 ± 0.02	0.50 ± 0.04
NS-3FO	1.31 ± 0.06	0.45 ± 0.04
CTRL	1.31 ± 0.05	0.43 ± 0.02

Values are the mean ± SEM (*n* = 6) for each group. * Significant difference (*p* < 0.05) compared to the CTRL group, using one-way ANOVA followed by the Tukey test.

**Figure 2.**
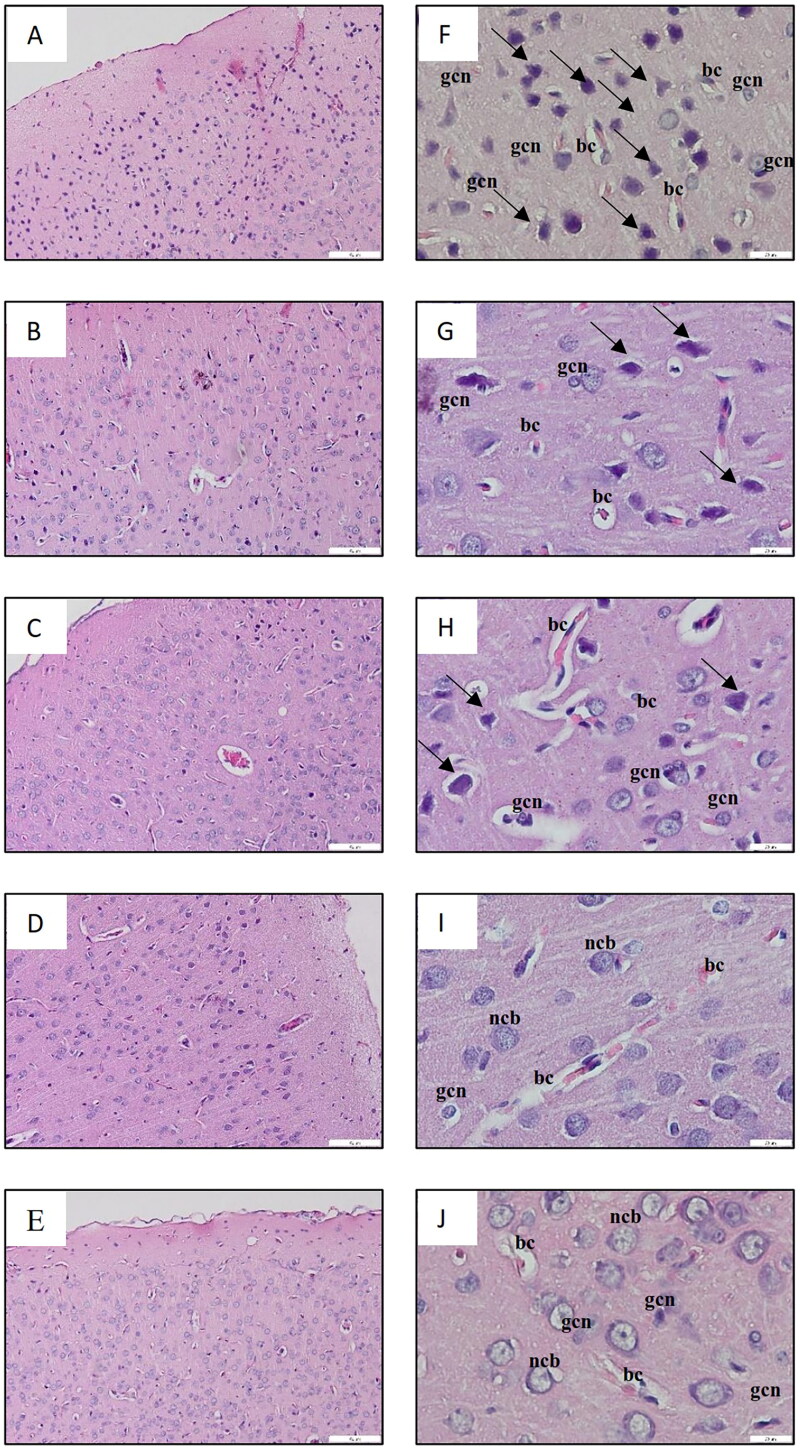
A-E Low magnification image of the cerebral cortex (10×, scale bar 200 µm), and F-J high magnification image of the cerebral cortex (40×, scale bar 20 µm) of HE staining from rat cerebrum. The DA-LPS group (F) shows scattered degenerating neurons with pyknotic nuclei, a large number of glial cells and dilated blood capillaries. The DA-LPS-1FO (G), and DA-LPS-3FO (H) showed fewer degenerating neurons with pyknotic nuclei (long arrow), a smaller number of glial cells, and slightly dilated blood capillaries. Meanwhile, the NS-3FO group (I) and CTRL group (J) showed no histopathological changes. ncb: neuron cell bodies; gcl: glial cell nuclei; bc: blood capilaries.

**Figure 3.**
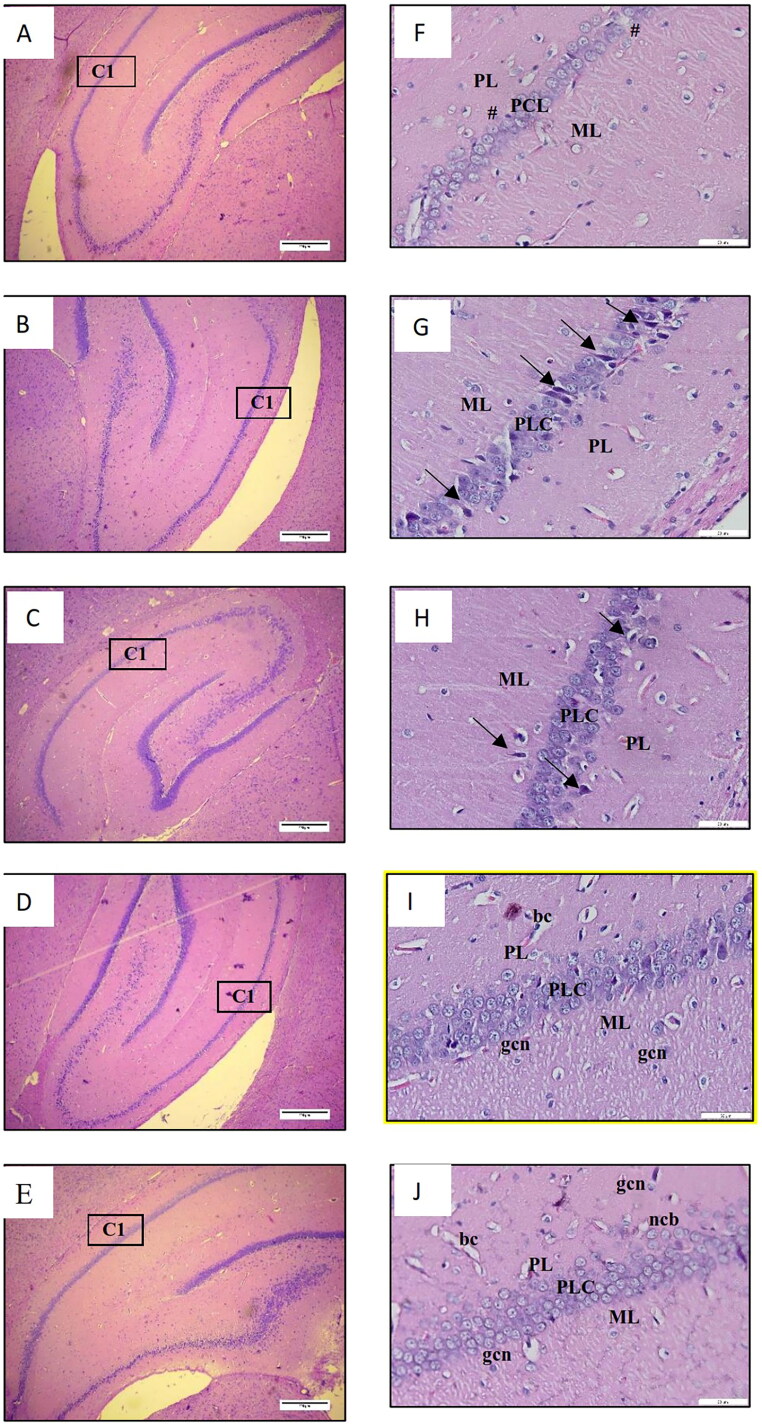
A-E Low magnification image of the hippocampus (4×, scale bar 200 µm) and F-J high magnification image of the hippocampus (20×, scale bar 50 µm) of HE staining from rat cerebrum. DA-LPS group (F) showed decreased of the PCL thickness (#). DA-LPS groups treated with 1 g/kg fish oil (G) and 3 g/kg fish oil (H) showed near normal thickness of the PCL with few degenerating neurons (black arrow). Meanwhile, the pyramidal neurons on the PCL formed a compact layer in the NA-3FO group (I) and the CTRL (J). ML: molecular layer, PCL: pyramidal cell layer, PL: Polymorphic layer, ncb: neuron cell bodies; gcl: glial cell nuclei; bc: blood capilaries.

Figure 2 displays the histological analysis of the cerebral cortex, while Figure 3 presents the observations of the hippocampal region referred to as cornu ammonis 1 (CA 1). The results of the histological examination revealed normal brain structure in both the NS-3FO and CTRL groups. The cerebral cortex exhibited dispersed nuclei consisting of both neurons and glial cells, whereas the CA 1 region of the hippocampus displayed 5-8 densely packed layers of pyramidal neurons in pyramidal cell layers (PCL). By contrast, the DA-LPS group exhibited noticeable neuronal necrosis in the cerebral cortex, characterized by the presence of dispersed degenerative neurons with pycnotic nuclei. This was followed by an increase in glial cells and dilatation of blood vessels. In a comparable manner, the CA 1 area of the hippocampus of DA-LPS group exhibited a reduction in the thickness of the PCL. Interestingly, the DA-LPS-1FO and DA-LPS-3FO exhibited a reduction in the number of deteriorated neurons and glial cells in the cortex. Additionally, in the CA 1 region, almost all pyramidal cell bodies were distributed normally, and the number of layers in the PCL approached the normal range.

### Effects of fish oil on the inflammatory mediators of diabetic animals with LPS induction

The inflammatory markers expression of TNF-α and IL-6 in different groups are presented in Table 4. The result showed that the inflammatory mediators of TNF-α and IL-6 in DA-LPS group was significantly higher (*p* < 0.001) compared to animals treated with 1 or 3 g/kg of fish oil supplementation (e.g., NS-3FO, DA-LPS-1FO and DA-LPS-3FO) and control group (CTRL).

**Table 4.** The Inflammatory markers expression of TNF-α and IL-6 in different groups.

Groups	TNF-α (% gated)	IL-6 (% gated)
DA-LPS	15.70 ± 3.3***	2.40 ± 0.65***
DA-LPS-1FO	3.78 ± 1.75	1.05 ± 0.26
DA-LPS-3FO	3.15 ± 0.42	1.21 ± 0.57
NS-3FO	3.72 ± 0.69	1.04 ± 0.19
CTRL	3.15 ± 0.98	1.27 ± 0.26

Values are the mean ± SEM (*n* = 6) for each group. *** Significant difference (*p* < 0.001) compared to the CTRL group, using one-way ANOVA followed by the Tukey test.

## Discussion

The current study shows that the combinatorial effects of STZ-induced hyperglycaemia and LPS-induced systemic inflammation in aggravating such effects. Diabetic animals with LPS-induced inflammation and treated with fish oil at either 1 or 3 g/kg for duration of 21 days had notably lower blood glucose levels. It has been established that fish oil administration showed an anti-hyperglycemic effect in both *in vivo* (Jangale et al. 2013; Parveen et al. 2019; Kobyliak et al. 2020) and *in vitro* (Laubertová et al. 2017; Das et al. 2022) studies. Indeed, fish oil has been found to possess antioxidant and anti-inflammatory properties (Erdogan et al. 2004; Purdel et al. 2021), which may help in preventing pancreatic histological alterations (Bellenger et al. 2011; Habib 2013), improving insulin production (Soltan 2012) and enhancing insulin sensitivity (Keapai et al. 2016). In addition, the present study demonstrated that diabetic rats receiving LPS-induced inflammation had a higher relative brain weight in comparison to the control groups. Presumably, the justification for this is that the hyperglycaemia generated by STZ may lead to significant weight loss as a result of insufficient insulin (Ventura-Sobrevilla et al. 2011; Hikmah et al. 2015; Samsulrizal et al. 2021), which triggers the breakdown of body fat and protein for energy production (Ghule et al. 2010). Previous study demonstrated that weight loss does not directly impact brain weight, but it does lead to a higher brain-to-body weight ratio (Sellers et al. 2007).

The development of hyperglycaemia and systemic brain inflammation complications in this study were evident from increasing cytokine pro-inflammation, altered brain tissue, and influence the cognitive performance in diabetic animals with LPS-induced inflammation. The current study discovered that diabetic animals with LPS-induced brain inflammation had higher concentrations of TNF-α and IL-6. This chronic inflammation in the brain ultimately impaired the animal’s cognitive function, as indicated by a reduced number of entries and time spent in the novel arm of the Y-maze test. Research has confirmed that prolonged inflammation in the brain caused by diabetes can activate many metabolic pathways, leading to impairments in cognitive abilities including as learning, memory, and spatial navigation (Gupta et al. 2023). Meanwhile, according to several studies, this memory impairment can also be caused by LPS injection (Zhu et al. 2014; Anaeigoudari et al. 2015; Zakaria et al. 2017). A study conducted on Sprague-Dawley diabetic rats revealed that the introduction of LPS caused a disturbance in insulin signaling, leading to an immediate inflammatory response and decreased spatial learning and memory (Murtishaw 2014).

Nevertheless, the present study found that diabetic animals receiving LPS induction and treated with fish oil supplementation had significantly lower levels of inflammation markers (e.g., TNF-α and IL-6) and improved cognitive performance in comparison to diabetic animals given LPS only. The cognitive performance was enhanced, as indicated by the increased number of entries in the novel arms relative to the other arms of the Y-maze test. Indeed, previous studies have mentioned that fish oil supplementation improves learning and memory performance in diabetic mice (Yang et al. 2012; Wang et al. 2020). This could indicate that cognitive impairment was closely related to brain inflammation due to the induction of LPS in diabetic rats. In addition, a study conducted on animal models utilizing diabetic and obese rats discovered a correlation between neuroinflammation and memory decline. The study observed a decrease in spatial memory and an increase in levels of pro-inflammatory cytokines (Dinel et al. 2011).

Physiologically, hyperglycaemia will activate the PKC pathway, which in turn activates the NF-kB pathway, resulting in neuroinflammation (Gupta et al. 2023). The findings indicated that fish oil, which contains higher levels of omega-3 fatty acids, has anti-inflammatory properties that can effectively suppress brain inflammation (Asari et al. 2019; Wang et al. 2020). Previous studies reported that omega-3 fatty acids can effectively suppress brain inflammatory responses by inhibiting microglial cell reactivity (Inoue et al. 2017; Gholamhosseinian et al. 2020). It is widely known that the overactivation of microglia and astrocytes will stimulate NF-ĸB molecules within the cells. This, in turn, results in the production of several cytotoxic substances, including superoxide radicals and pro-inflammatory mediators such as TNF-α, IL-1, and IL-6 (Kwon and Koh 2020; Rauf et al. 2022). Yet, the omega-3 fatty acids modulate microglial cells by inhibiting the activation of p38 MAPK (Lu et al. 2013), reducing the production of inflammatory molecules, and activating peroxisome proliferator-activated receptor γ (PPARγ) (Ajmone-Cat et al. 2012).

Finally, our research confirmed that the administration of fish oil at doses of 1 and/or 3 g/kg in diabetic animals with LPS-induced inflammation effectively decreased the migration of glial cells and the degeneration of neurons in the cerebral cortex of the brain. The same outcomes were also observed in the hippocampus, a region of the brain that has been extensively studied for its role in memory and learning (Anand and Dhikav 2012). The CA 1, which is the biggest region in the hippocampus, is commonly employed as a model system for investigating plasticity, pharmacological effects, and intracellular characteristics (Mizuseki et al. 2011). The histological examination of the CA 1 region revealed less severe neuronal damage in diabetic animals that received fish oil supplementation together with LPS, compared to diabetic animals which received LPS without fish oil supplementation. This study believed that the Y-maze outcome, which demonstrated an improvement in the rat’s spatial learning and memory, is directly related to the ameliorative role of fish oil in preventing brain tissue damage. Moreover, this study assumed that the histological findings were strongly correlated with the increasing levels of pro-inflammatory mediators in diabetic animals receiving LPS induction but without fish oil supplementation. Diabetes has the ability to release pro-inflammatory mediators that can modify the brain’s structure (Gaspar et al. 2016). Furthermore, the increase in pro-inflammatory mediators may provoke and intensify neurodegeneration, resulting in tissue damage (Glass et al. 2010; Konsman 2022). Taken together, the histopathological findings in this study support previous research (Afshordel et al. 2015) that claims fish oil rich in omega-3 fatty acids provides neuroprotection. Indeed, beyond its well-known anti-inflammatory and neuroprotective effects, omega-3 fatty acids also prolong neuronal cell longevity, reduce neurodegeneration, and prevent cognitive impairment (Kim 2014).

## Conclusions

This study highlights the potential of menhaden fish oil in mitigating hyperglycaemia, neuroinflammation, brain morphological changes, and cognitive dysfunction in diabetic rats induced with LPS. Thus, our findings promote the regular intake of fish oil as an attempt to preserve brain health and function to avoid the cognitive impairment associated with diabetes. Nonetheless further clinical trial research is required to provide additional evidence for the beneficial effects of fish oil in preventing diabetic brain disruption.
